# Effect of endurance training and cinnamon supplementation on post-exercise oxidative responses in rats

**Published:** 2014-12

**Authors:** Gholamreza Dehghan, Mehrnoosh Shaghaghi, Afshar Jafari, Mustafa Mohammadi, Reza Badalzadeh

**Affiliations:** 1 Department of Biology, Faculty of Natural Science, University of Tabriz, Tabriz, Iran; 2 Department of Biology, Faculty of Basic Science, Tehran Payamenoor University, Tehran, Iran; 3 Department of Sports Physiology, Faculty of Physical Education and Sports Sciences, University of Tabriz, Tabriz, Iran; 4 Department of Physiology, Drug Applied Research Center, Tabriz University of Medical Sciences, Tabriz, Iran

**Keywords:** Endurance training, oxidative stress, exhaustive exercise, lipid peroxidation

## Abstract

Despite the preventative and therapeutic effects of regular exercise, exhaustive exercise may be harmful to health. The present study aimed to determine the protective effect of endurance training and cinnamon bark extract (CBE) supplementation on oxidative responses induced by an exhaustive exercise schedule in rats. The rats were randomly divided into the following five groups of 6; control sedentary (Con/Sed), control exercised (Con/Ex), trained exercised (Tr/Ex), supplemented exercised (Sup/Ex), and trained, supplemented and exercised (Tr/Sup/Ex). Animals in exercise groups ran on a rodent treadmill for an 8-week endurance training program. At the end of the experiment, blood samples were collected and (MDA) and total thiol (TT) levels were measured in plasma. Glutathione peroxidase (GPX), superoxide dismutase (SOD), and catalase (CAT) activities were determined in soleus muscles. Results showed significant increases in SOD activity and malondealdehyde (MDA) levels in the soleus muscles and serum of exercised rats fed with the normal diet. The exhaustive exercise also induced a decrease in serum total thiol level and GPX activity. Elevated levels of total thiol and total antioxidant capacity (TAC) and reduced serum MDA levels were found in the Sup/Ex and Tr/Sup/Ex groups. CAT and GPX activities increased by CBE treatment in trained rats. Regular training increased CAT and GPX activities in the Tr/Sup/Ex group. CAT, GPX and SOD activities were not affected by the CBE treatment in untrained rats. Results suggest that additional use of regular training and CBE supplementation increase TAC and protect healthy male rats against oxidative damage induced by exhaustive exercise.

## INTRODUCTION

Preventative and therapeutic effects of regular, non-exhaustive exercise on chronic diseases such as diabetes, cancer, hypertension, cardiovascular diseases, osteoporosis, depression and obesity have been demonstrated by previous research. However, exhaustion can destroy the useful effects of exercise [1, 2]. During exhaustive exercise, the body's oxygen consumption and oxygen uptake markedly increase through the muscles, inducing harmful effects on health. This causes an accelerated generation of reactive oxygen species (ROS) to exceed the capacity of body antioxidant defenses known as oxidative stress [1, 3]. Extra accumulation of ROS results in structural damage to the contractile tissue, especially skeletal muscles, by oxidizing cellular components such as membrane lipids, proteins, carbohydrates and DNA [4]. Previous studies have reported that by-products of lipid peroxidation, especially malondyaldehyde (MDA), increase following a single bout of exhaustive exercise [4-6]. Compared to control groups, exhausted subjects have also shown reduced glutathione levels (GSH) in the liver, skeletal muscles and plasma [7, 8]. In mammalian tissues, oxidative damage induced by free radicals is prevented by complex antioxidant systems. These systems include enzymes such as superoxide dismutase (SOD), catalase (CAT) and glutathione peroxidase (GPX) and an array of small molecules, including ascorbic acid, alpha-tocopherol, ubiquinol-10, reduced glutathione (GSH), methionine, uric acid, and bilirubin [9, 10]. Previous studies have shown that lipid peroxidation decreased after exhaustive exercise in trained as compared to controlled subjects. It has been proposed that regular moderate exercise may protect subjects against exercise induced oxidative stress by increasing the expression of antioxidant enzymes [11, 12]. 

Cinnamon (*Cinnamomum zeylanicum*) has been used as popular condiment in human food. The excellent antioxidant activities of cinnamon bark, leaf and fruit extracts and essential oils have been reported in several in vitro and *in vivo* studies [13-18]. 

The aim of this study was to determine the protective effects of cinnamon bark extract (CBE) supplementation and endurance training in attenuating markers of oxidative stress induced by exhaustive exercise in trained and untrained male rats. Consequently, oxidative stress markers such as serum and soleus muscles' MDA concentration, total thiol (TT) serum level and GPX, CAT, and SOD activities in soleus muscles and serum TAC were measured.

## MATERIALS AND METHODS

**Experimental design: Experimental design: **Thirty 8-week-old male Wistar rats (250-300 g) were housed in a clean rodent room under a 12:12-h light-dark cycle (07:00-19:00 h dark: 19:00-07:00 h light), at a temperature of 24 ± 1C. The animals were fed a standard rodent laboratory diet and tap water *ad libitum*. Animal experimentations were approved by the Ethical Committee of Tabriz University of Medical Sciences and carried out in an ethically proper way by following the provided guidelines. The rats were divided into five groups (n=6) based on exhaustive exercise, regular aerobic training (RT) and CBE supplementation. The groups included Con/Sed (control sedentary with normal diet) Con/Ex (normal diet with exhaustive exercise in last session), Tr/Ex (regularly trained for 8 weeks with normal diet and ran on treadmill to exhaustion in last session), Sup/Ex (supplemented with CBE for 8 weeks and exhaustive exercise in last session) and Tr/Sup/Ex (regularly trained and supplemented with CBE for 8 weeks and ran on treadmill to exhaustion in last session). All rats were familiarized with treadmill running for one week at 10 m/min, 0% grade for 10 min/day. Then, an 8-week endurance training program began with progressive physical exercise during which training speed and time gradually increased to 22 m/min for 90 min/day at week 4. Training continued 90 min/day, 5 days/week for 8 weeks. In the last session, trained and untrained rats ran on the treadmill to exhaustion; running speed began with 10 m/min, progressively increasing to 22 m/min at the 12^th^ min and kept fixed thereafter to make rats exhausted [19]. Rats in Sup/Ex and Tr/Sup/Ex groups received 200 mg/kg/day of CBE for 8 weeks [20], and were immediately anesthetized by ketamine infusion (60 mg/kg) and xylazine (10 mg/kg). After surgery, blood samples, collected from portal veins, were centrifuged at 2000 g for 10 min at 4°C. The plasma was kept at 20C for subsequent determination of lipid peroxidation and antioxidant status. Soleus muscles were removed from both hind limbs and dissected from fat and connective tissue. The muscles were homogenized in KCl (1.15%) using a glass-Teflon homogenizer on ice. The homogenates were centrifuged at 1500 g for 20 min, and supernatants were collected for biochemical analysis. The resulting supernatant was stored at -80 ºC until use. 

**Preparation of cinnamon bark extract: **Cinnamon barks were powdered, and 350 g were extracted five times with methanol (MtOH) (90%) overnight at room temperature and dried using a vacuum evaporator. Extracts (50 g) were stored at -20 ºC until use. The extract was dissolved in carboxymethyl cellulose (CMC) 0.5% for *in vivo* use*,*

**Analytical methods: **As an index of lipid peroxidation in soleus muscles and serum, MDA concentration was determined by measuring the TBARS (Thiobarbituric Acid Reactive Substances) formed during an acid-heating reaction [21]. Briefly, 1 ml of homogenate or serum was mixed with 4 ml of 20% trichloroacetic acid (TCA) and 1 ml of 0.5% thiobarbituric acid, heated in a boiling water bath for 30 min and immediately cooled on ice. Samples were then centrifuged at 1000 g for 15 min at 4 ºC. TBARS were determined by absorbance at 535 nm. Results were expressed as TBARS concentration per milligram of protein. Total serum antioxidant capacity was determined using a ferric reducing ability of plasma (FRAP) test and 2, 4, 6-tripyridyl-s-triazine (TPTZ) as reagent [22]. Total serum thiols (TT) concentration was determined as an index of protein oxidation. Plasma TT were measured using a spectrophotometric assay at 412 nm using DTNB as reagent [23]. 

**Determination of GPX, CAT, SOD activities in soleus muscle: **GPX and SOD activities in muscle tissues were measured by related kits (Randox Co Germany). GPX activity was determined at 340 nm by a spectrophotometer as described by *Paglia* and *Valentine* (1967). This method is based on NADPH oxidation in the presence of H_2_O_2_ as substrate [24]. SOD activity was measured as described by *Delmas-Beauviex* (1995). This method is based on the reduction of nitroblue tetrazolium in a xanthine- oxidase-dependent superoxide generation system. One unit of SOD was defined as the amount of enzyme required to induce a 50% inhibition of NBT and the specific enzyme activity was expressed as unites per milligrams of protein [25]. Catalase activity in soleus muscles was detected by measuring H_2_O_2 _decomposition according to the Aebi method [26]. The reaction was started by adding H_2_O_2_, and absorbance was measured at 240 nm. Catalase activity was expressed as the decrease in H_2_O_2_ nanomoles per minute per milligram of protein.

**Statistical analysis: **Statistical analysis of data was carried out using SPSS 16 for windows software (SPSS INC, Chicago, IL, USA). The sample size was 6 for each group. One-way ANOVAs with post-hoc multicomparison tests were used to compare group means. P values less than 0.05 were considered significant. Data were expressed as means±SD. 

Thirty 8-week-old male Wistar rats (250-300 g) were housed in a clean rodent room under a 12:12-h light-dark cycle (07:00-19:00 h dark: 19:00-07:00 h light), at a temperature of 24 ± 1C. The animals were fed a standard rodent laboratory diet and tap water *ad libitum*. Animal experimentations were approved by the Ethical Committee of Tabriz University of Medical Sciences and carried out in an ethically proper way by following the provided guidelines. The rats were divided into five groups (n=6) based on exhaustive exercise, regular aerobic training (RT) and CBE supplementation. The groups included Con/Sed (control sedentary with normal diet) Con/Ex (normal diet with exhaustive exercise in last session), Tr/Ex (regularly trained for 8 weeks with normal diet and ran on treadmill to exhaustion in last session), Sup/Ex (supplemented with CBE for 8 weeks and exhaustive exercise in last session) and Tr/Sup/Ex (regularly trained and supplemented with CBE for 8 weeks and ran on treadmill to exhaustion in last session). All rats were familiarized with treadmill running for one week at 10 m/min, 0% grade for 10 min/day. Then, an 8-week endurance training program began with progressive physical exercise during which training speed and time gradually increased to 22 m/min for 90 min/day at week 4. Training continued 90 min/day, 5 days/week for 8 weeks. In the last session, trained and untrained rats ran on the treadmill to exhaustion; running speed began with 10 m/min, progressively increasing to 22 m/min at the 12^th^ min and kept fixed thereafter to make rats exhausted [19]. Rats in Sup/Ex and Tr/Sup/Ex groups received 200 mg/kg/day of CBE for 8 weeks [20], and were immediately anesthetized by ketamine infusion (60 mg/kg) and xylazine (10 mg/kg). After surgery, blood samples, collected from portal veins, were centrifuged at 2000 g for 10 min at 4°C. The plasma was kept at 20C for subsequent determination of lipid peroxidation and antioxidant status. Soleus muscles were removed from both hind limbs and dissected from fat and connective tissue. The muscles were homogenized in KCl (1.15%) using a glass-Teflon homogenizer on ice. The homogenates were centrifuged at 1500 g for 20 min, and supernatants were collected for biochemical analysis. The resulting supernatant was stored at -80 ºC until use. 

**Preparation of cinnamon bark extract: **Cinnamon barks were powdered, and 350 g were extracted five times with methanol (MtOH) (90%) overnight at room temperature and dried using a vacuum evaporator. Extracts (50 g) were stored at -20 ºC until use. The extract was dissolved in carboxymethyl cellulose (CMC) 0.5% for *in vivo* use*,*

**Analytical methods: **As an index of lipid peroxidation in soleus muscles and serum, MDA concentration was determined by measuring the TBARS (Thiobarbituric Acid Reactive Substances) formed during an acid-heating reaction [21]. Briefly, 1 ml of homogenate or serum was mixed with 4 ml of 20% trichloroacetic acid (TCA) and 1 ml of 0.5% thiobarbituric acid, heated in a boiling water bath for 30 min and immediately cooled on ice. Samples were then centrifuged at 1000 g for 15 min at 4 ºC. TBARS were determined by absorbance at 535 nm. Results were expressed as TBARS concentration per milligram of protein. Total serum antioxidant capacity was determined using a ferric reducing ability of plasma (FRAP) test and 2, 4, 6-tripyridyl-s-triazine (TPTZ) as reagent [22]. Total serum thiols (TT) concentration was determined as an index of protein oxidation. Plasma TT were measured using a spectrophotometric assay at 412 nm using DTNB as reagent [23]. 

**Determination of GPX, CAT, SOD activities in soleus muscle: **GPX and SOD activities in muscle tissues were measured by related kits (Randox Co Germany). GPX activity was determined at 340 nm by a spectrophotometer as described by *Paglia* and *Valentine* (1967). This method is based on NADPH oxidation in the presence of H_2_O_2_ as substrate [24]. SOD activity was measured as described by *Delmas-Beauviex* (1995). This method is based on the reduction of nitroblue tetrazolium in a xanthine- oxidase-dependent superoxide generation system. One unit of SOD was defined as the amount of enzyme required to induce a 50% inhibition of NBT and the specific enzyme activity was expressed as unites per milligrams of protein [25]. Catalase activity in soleus muscles was detected by measuring H_2_O_2 _decomposition according to the Aebi method [26]. The reaction was started by adding H_2_O_2_, and absorbance was measured at 240 nm. Catalase activity was expressed as the decrease in H_2_O_2_ nanomoles per minute per milligram of protein.

**Statistical analysis: **Statistical analysis of data was carried out using SPSS 16 for windows software (SPSS INC, Chicago, IL, USA). The sample size was 6 for each group. One-way ANOVAs with post-hoc multicomparison tests were used to compare group means. P values less than 0.05 were considered significant. Data were expressed as means±SD.

## RESULTS AND DISCUSSION

The MDA level in soleus muscles for the Con/Ex group was significantly higher than that of the Con/Sed group (p<0.05). MDA concentration in soleus muscles for the Sup/Ex group did not significantly differ from that of Con/Sed and Con/Ex groups (table 1).

**Table 1:** Effect of 8-week regular training and supplementation with 200 mg/kg of CBE on antioxidant enzyme activity and MDA level changes in soleus muscle among five groups of male rats submitted to an exhaustive exercise

**Groups**	**CAT** (nmol/min/mg protein)	**GPX** (U/mg protein)	**SOD ** (U/mg protein)	**MDA** (nmol/mg protein)
**Sup/Ex**	14.83 ± 2.17	0.56 ± 0.064	7.473 ± 0.69	0.061±0.007
**Con/Ex**	14.98 ± 1.14	0.412 ± 0.077*	8.431 ± 0.6*	0.065± 0.01*
**Con/Sed**	12.96 ± 2.18	0.722 ± 0.082	7.257 ± 0.49	0.045±0.01
**Tr/Ex**	0.06± 0.01	21.93± 3.27 * #	0.59± 0.08 #	8.36± 0.85
**Tr/Sup/Ex**	0.06± 0.01	22.86± 1.5 * #	0.60± 0.07 #	8.09± 0.64

Result are expressed as mean ± SD (n=6). Abbreviations; MDA: malondialdehyde; CAT: catalase; GPX: glutathione peroxidase; SOD: superoxide dismutase. Differences of p<0.05 were considered significant. Significant differences:

* Con/Ex, Tr/Ex and Tr/Sup/Ex vs. Con/Sed;

# Tr/Ex and Tr/Sup/Ex vs. Con/Ex

**Figure 1:**
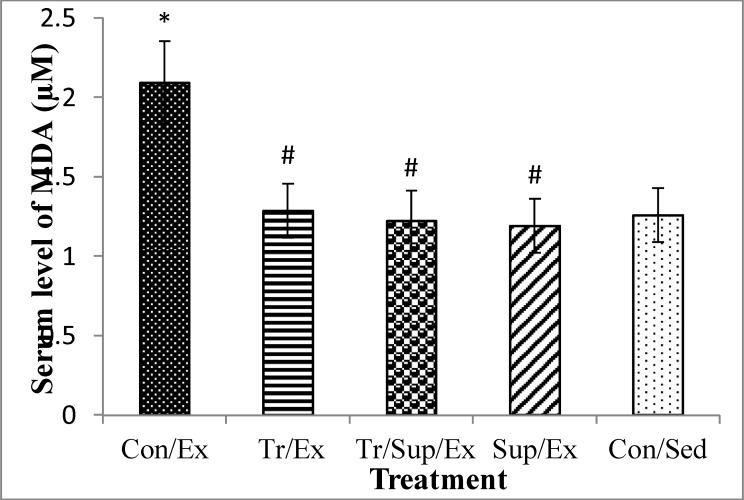
Effect of 8-week regular training and supplementation with 200 mg/kg of CBE on serum MDA content in healthy male rats submitted to an exhaustive exercise.

As figure 1 shows, the concentration of plasma MDA, a lipid peroxidation index, in the Sup/Ex (1.2± 0.24) and Con /Sed (1.25± 0.17) groups also decreased significantly (P<0.05). 

Result are expressed as mean ± SD (n=6 for each group). Difference between Con/Ex and other groups is significant at: (^#^) p< 0.05 and (^*^) p< 0.01. Arrangement: control sedentary (Con/Sed), control exercised (Con/Ex), trained exercised (Tr/Ex), supplemented exercised (Sup/Ex), trained, supplemented and exercised (Tr/Sup/Ex)

**Figure 2:**
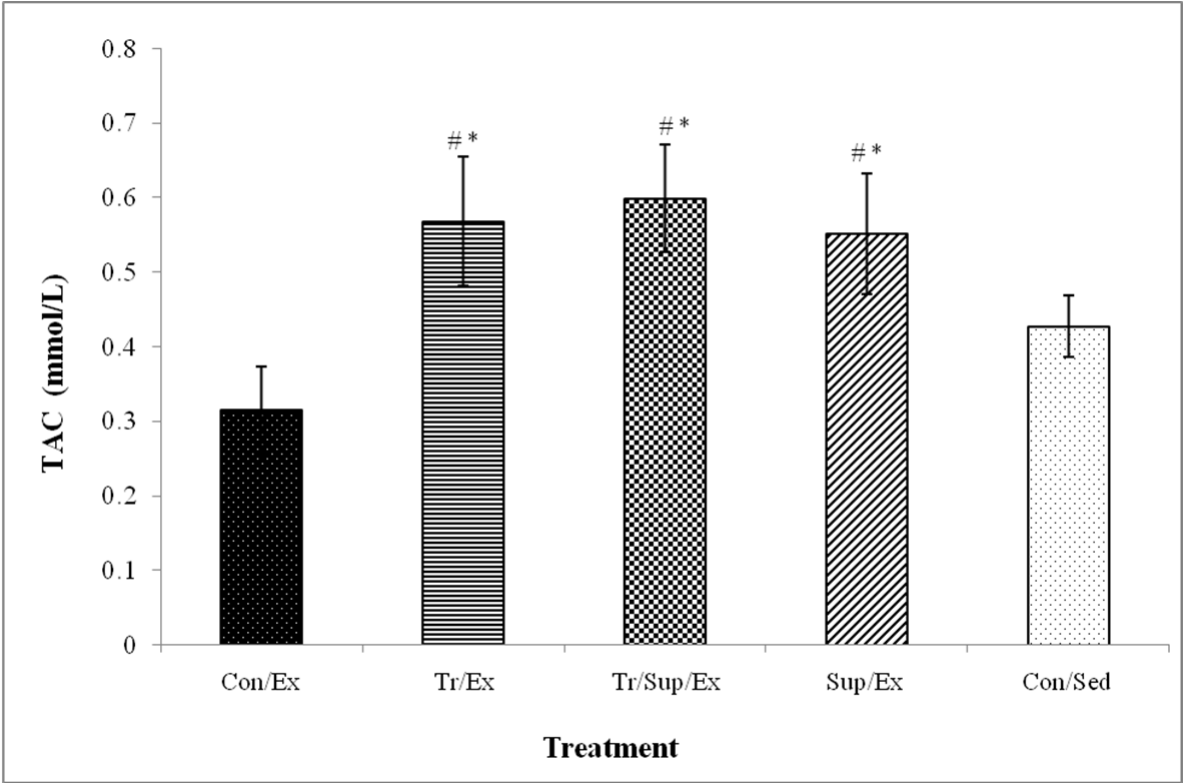
Effect of 8-week regular training and supplementation with 200 mg/kg of CBE on serum total antioxidant capacity (TAC) in healthy male rats submitted to an exhaustive exercise

The alteration of plasma total antioxidant capacity following exhaustive exercise in the Con/Ex group (0.552±0.08) was not significant as compared to the Con/Sed group (0.43± 0.04). In the Sup/Ex group, supplementation with CBE significantly increased serum total antioxidant capacity (0.32± 0.06) compared to Con/Ex group (p<0.01) (figure 2). 

Result are expressed as mean ± SD (n=6 for each group). Difference between treated groups and Con/Ex (^#^) and Con/Sed (^*^) are significant at p < 0.05.

**Figure 3:**
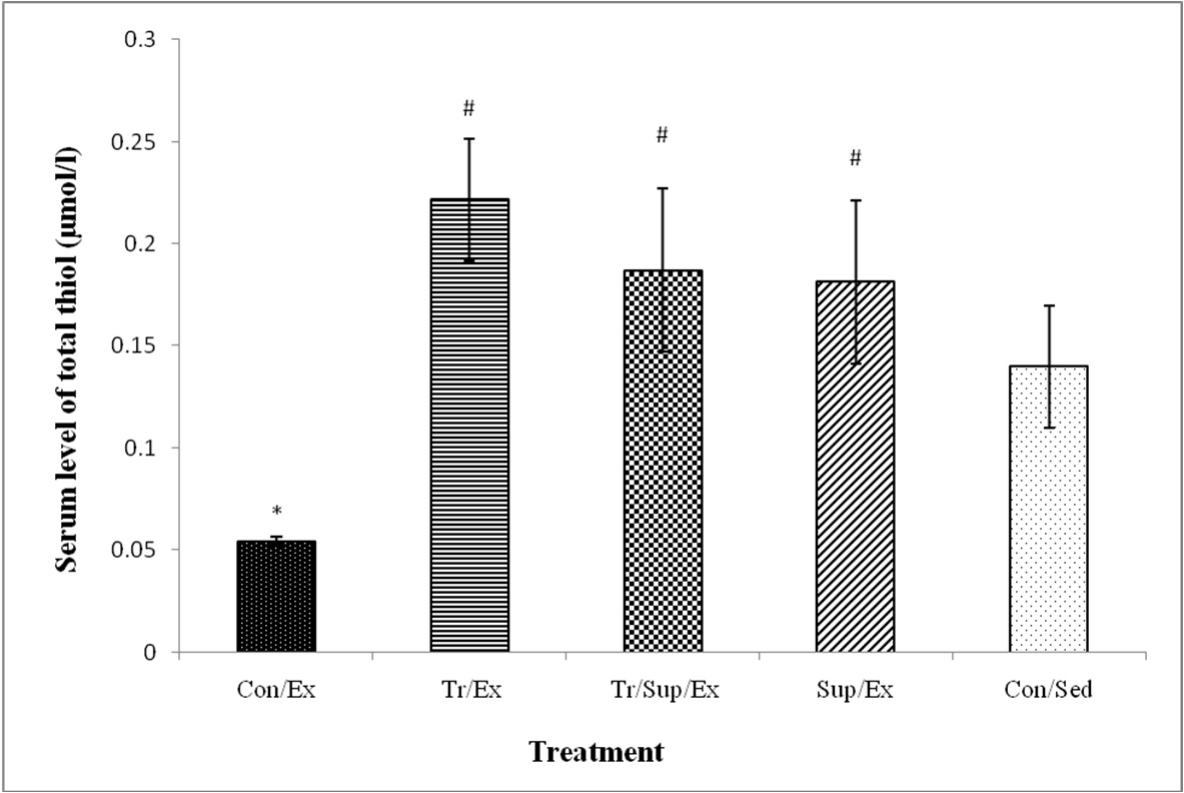
Effect of 8-week regular training and supplementation with 200 mg/kg of CBE on serum total thiol content in healthy male rats submitted to an exhaustive exercise.

Plasma total thiol concentration for the Con/Ex group (0.05± 0.003) was significantly lower than that of the Con/Sed group (0.11±0.01) (p<0.01). As figure 3 shows, Supplementation with CBE significantly increased serum total thiol in the Sup/Ex group (0.16± 0.01) compared to the Con/Sed and Con/Ex groups (p<0.01).

Result are expressed as mean ± SD (n=6 for each group). Difference between Con/Ex treated groups (^#^) and Con/Ex vs. Con/Sed (^*^) are significant at p < 0.05.

Acute exhaustive exercise significantly decreased GPX activities in the soleus muscles of the Con/Ex group compared to the Con/Sed group (p<0.01). GPX activity in the soleus muscle for the Sup/Ex group did not significantly differ from those of Con/Ex and Con/Sed groups (table 1). SOD activity in soleus muscles of the Con/Ex group was significantly higher than that of the Con/Sed group (p<0.05). SOD activity in the soleus muscles of the Sup/Ex group did not significantly differ from those of Con/Sed and Con/Ex groups (table 1). CAT activity in soleus muscles of Sup/Ex and Con/Ex groups did not significantly differ from that of the Con/Sed group (table 1). 

As figure 1 shows, significant decreases in MDA serum levels in Sup/Ex (1.2± 0.24), Tr/Ex (1.28± 0.17), Tr/Sup/Ex(1.22± 0.19) and Con/Sed (1.25± 0.17) groups were observed compared to the Con/Ex group (2.09± 0.26) (p<0.01).

MDA level in the soleus muscle for the Con/Ex group was significantly higher than that of the Con/Sed group (p<0.05). MDA concentrations in soleus muscles of Sup/Ex, Tr/Ex and Tr/Sup/Ex groups did not significantly differ from those of Con/Sed and Con/Ex groups (table 1).

Decrease of serum total antioxidant capacity following exhaustive exercise in the Con/Ex group (0.552±0.081) compared to the Con/Sed group (0427±0.041) was not significant. As figure 2 shows, supplementation with CBE and RT performance significantly increased serum total antioxidant capacity in Sup/Ex (0.315±0.059), Tr/Ex (0.56±0.08) and Tr/Sup/Ex(0.59±0.08) groups compared to Con/Ex and Con/Sed groups (p<0.01) 

Serum total thiol (TT) concentration for the Con/Ex group (0.054±0.002) was significantly lower than that of the Con/Sed group (0.14±0.03) (p<0.05). As figure 3 shows, supplementation with CBE and RT significantly increased serum total thiol in the Sup/Ex (0.18±0.04), Tr/Ex (0.22±0.03) and Tr/Sup/Ex (0.18±0.04) groups compared to the Con/Ex group (p<0.01).

Acute exhaustive exercise significantly decreased GPX activities in the soleus muscles of the Con/Ex group compared to the Con/Sed group (p<0.01). GPX activity in the soleus muscles of the Sup/Ex group did not significantly differ from those of the Con/Ex and Con/Sed groups. As table 1 shows, Increase of GPX activity in soleus muscles of the Tr/Ex and Tr/Sup/Ex groups was significant as compared to the Con/Ex group (p<0.05). 

SOD activity in the soleus muscles of the Con/Ex group was significantly higher than that of the Con/Sed group (p<0.05). SOD activity in the soleus muscles of Sup/Ex, Tr/Ex and Tr/Sup/Ex groups did not significantly differ from those of Con/Sed and Con/Ex groups (table 1). CAT activity in soleus muscles for Sup/Ex and Con/Ex groups did not significantly vary from that of the Con/Sed group. As table 1 indicates, increase of CAT activity in Tr/Ex and Tr/Sup/Ex groups was significant compared to Con/Sed and Con/Ex groups (p< 0.01).

Oxidative damage in these animals may be due to the free radicals produced by exhaustive exercise. Elevated MDA concentration and decrease of total thiol values after exhaustive exercise is usually expected. Hessel et.al (2000) reported an apparent elevation in MDA and GSSG serum levels after running marathons [4]. 

This study demonstrated that as symptoms of oxidative damage, MDA levels in soleus muscles and serums significantly increased in the Con/Ex group after exhaustive exercise. Also, there was significant decrease in serum total thiol values in the Con/Ex group following exhaustive exercise. Aguilo et.al (2005) reported increases in blood GSSG after mountain cycling [7]. Huang et.al (2008) and Miazaki et.al (2001) reported increased lipid peroxidation in muscular tissues caused by acute exhaustive exercise [27, 28]. Similar to other studies, our results show that oxidative damage induced by lipid peroxidation can be attributed decreased GSH levels. During acute exercise, cellular NADPH concentration reduces following increased muscular metabolism. Thus, reducing GSSG to GSH may not be sufficient. Regular exercise has been shown to improve antioxidant defenses and may decrease oxidative stress induced by acute exercise [11, 12, 28]. In the present study, serum MDA concentration decrease, total thiol level increase and TAC were observed in trained rats. Moreover, MDA concentrations in soleuse muscles were not affected by aerobic training in the trained rats. As a functional sulfhydryl (SH), thiol content change is the main determinant of TAC changes; it can also be considered as an index of protein oxidation in tissues and plasma. Similar to our study, regular training has been reported to increase plasma total thiol in the young soccer players [30]. Increases of GSH during regular training may due to upregulation of gamma glutamyl transferase. Many researchers have reported that antioxidant nutrient intake attenuates oxidative stress in response to acute exercise. Some fruits and vegetables contain several antioxidant nutrients including vitamins, flavonoids and polyphenolic compounds. In the current study, supplementing healthy rats with CBE decreased oxidative damage induced by exhaustive exercise as indicated by MDA serum level and total thiol in Sup/Ex group. Elevated TAC for the Sup/Ex group showed that CBE supplementation increased the amount of antioxidants available to the animals. Several studies have indicated that cinnamon has protective effects against many oxidative stress related diseases in humans [19, 21]. Ranjbar et.al (2006) maintained that the regular consumption of cinnamon tea decreased lipid peroxidation and increased TAC in human subjects [18]. In a study by Lee et.al (2003) it was shown that the elevation of antioxidant defense in high cholesterol-fed rats was subject to cinnamon supplementation [17]. Moselhy and Junbi (2010) reported that cinnamon extract has hepatoprotective effects against CCl_4_-induced toxicity by decreasing lipid peropxidation and enhancing antioxidant enzyme activities in rats [20]. Many studies have reported extracts from bark, leaves and fruits of cinnamon to have high phenol contents and excellent potential in scavenging free radicals [14, 16]. Such activity may be due to the existence of phenolic hydroxyl groups in the eugenol which is present in cinnamon [15]. In a study by Mashhadi et. al (2013)it was shown that cinnamon and ginger decreased MDA concentrations in female athletes [29]. Simoes et.al (2008) reported increased TSH levels and serum TAC in subjects supplemented with green tea during exhaustive exercise [31]. Dunlap et. al (2006) reported the elevation of total antioxidant power in sled dogs supplemented with blueberries [32]. A study by Morillas- Ruiz et.al (2006) also showed that using polyphenol compounds in cyclists decreased lipid peroxidation in response to intense exercise [33]. 

 Several studies have reported inconsistent changes of antioxidant activities during exercise in various tissues [34- 37]. In this study, exhaustive exercise was found to elevate SOD activity and decrease GPX activity in soleus muscles. Moreover, CAT activity in soleus muscles was not affected by acute exhaustive exercise. In our study, the decrease of GPX activity in soleus muscles caused by acute exhaustive exercise in untrained rats was prevented by aerobic training and CBE supplementation in the Tr/Ex and Tr/Sup/Ex groups. Aerobic training also increased CAT activity in the Tr/Ex and Tr/Sup/Ex groups, but SOD activity was not affected by aerobic training and CBE supplementation. Other studies have also reported that GPX, CAT and SOD activities increased after aerobic exercise training in rats [38, 39]. Increase of oxygen radicals following acute exhaustive exercise could induce proteolysis of GPX. Increase of SOD activity during acute exhaustive exercise could also be induced by the elevation of superoxide radicals as enzyme substrates. Increase of ROS generation during exercise training may activate adaptive responses to oxidative stress by upregulating antioxidant enzyme gene expressions. Therefore, various defense responses to exercise-induced oxidative stress can be caused by differences in exercise intensity and duration, antioxidant status, age of subjects and the measurement time of biomarkers. In this study, supplementing healthy male rats with CBE did not change antioxidant enzyme activity (SOD, CAT, and GPX) in soleus muscles associated with exhaustive exercise. This might have resulted from the high potential of cinnamon in scavenging free radicals induced by exercise. Thus, in our study, CBE has possibly acted as a potent antioxidant by scavenging free radicals.

To summarize, we showed that long term CBE supplementation attenuated oxidative damage induced by acute exhaustive exercise by increasing the total antioxidant power in trained and untrained rats.

## Notes

**Acknowledgements: **This research was supported by grants from the Drug Applied Research Center, Tabriz University of Medical Sciences, Tabriz, Iran.
